# Refocusing of Attention on Positive Events Using Monitoring-Based Feedback and Microinterventions for Patients With Chronic Musculoskeletal Pain in the PerPAIN Randomized Controlled Trial: Protocol for a Microrandomized Trial

**DOI:** 10.2196/43376

**Published:** 2023-09-20

**Authors:** Leonie Ader, Anita Schick, Martin Löffler, Annette Löffler, Eva Beiner, Wolfgang Eich, Stephanie Vock, Andrei Sirazitdinov, Christopher Malone, Jürgen Hesser, Michael Hopp, Christian Ruckes, Herta Flor, Jonas Tesarz, Ulrich Reininghaus

**Affiliations:** 1 Department of Public Mental Health, Central Institute of Mental Health, Medical Faculty Mannheim, Heidelberg University Mannheim Germany; 2 Institute of Cognitive and Clinical Neuroscience, Central Institute of Mental Health, Medical Faculty Mannheim, Heidelberg University Mannheim Germany; 3 Department of General Internal Medicine and Psychosomatics, Heidelberg University Heidelberg Germany; 4 Data Analysis and Modeling, Mannheim Institute for Intelligent Systems in Medicine, Medical School Mannheim, Heidelberg University Mannheim Germany; 5 Central Institute for Scientific Computing, Heidelberg University Heidelberg Germany; 6 Central Institute for Computer Engineering, Heidelberg University Heidelberg Germany; 7 CZS Heidelberg Center for Model-Based AI, Heidelberg University Heidelberg Germany; 8 Interdisciplinary Center for Clinical Trials, Johannes Gutenberg University Medical Center Mainz Germany; 9 Centre for Epidemiology and Public Health, Health Service and Population Research Department, Institute of Psychiatry, Psychology & Neuroscience, King’s College London London United Kingdom; 10 ESRC Centre for Society and Mental Health, King´s College London London United Kingdom

**Keywords:** experience sampling method, ESM, ecological momentary intervention, EMI, microrandomized trial, mobile health, mHealth, positive intervention, complex intervention, mobile phone

## Abstract

**Background:**

Chronic musculoskeletal pain (CMSP) affects between 13% and 47% of the population, with a global growth rate of 20.3% within the last 15 years, suggesting that there is a high need for effective treatments. Pain diaries have long been a common tool in nonpharmacological pain treatment for monitoring and providing feedback on patients’ symptoms in daily life. More recently, positive refocusing techniques have come to be used, promoting pain-free episodes and positive outcomes rather than focusing on managing the pain.

**Objective:**

This study aims to evaluate the feasibility (ie, acceptability, intervention adherence, and fidelity) and initial signals of efficacy of the PerPAIN app, an ecological momentary intervention for patients with CMSP. The app comprises digitalized monitoring using the experience sampling method (ESM) and feedback. In addition, the patients receive 3 microinterventions targeted at refocusing of attention on positive events.

**Methods:**

In a microrandomized trial, we will recruit 35 patients with CMSP who will be offered the app for 12 weeks. Participants will be prompted to fill out 4 ESM monitoring questionnaires a day assessing information on their current context and the proximal outcome variables: absence of pain, positive mood, and subjective activity. Participants will be randomized daily and weekly to receive no feedback, verbal feedback, or visual feedback on proximal outcomes assessed by the ESM. In addition, the app will encourage participants to complete 3 microinterventions based on positive psychology and cognitive behavioral therapy techniques. These microinterventions are prompts to report joyful moments and everyday successes or to plan pleasant activities. After familiarizing themselves with each microintervention individually, participants will be randomized daily to receive 1 of the 3 exercises or none. We will assess whether the 2 feedback types and the 3 microinterventions increase proximal outcomes at the following time point. The microrandomized trial is part of the PerPAIN randomized controlled trial (German Clinical Trials Register DRKS00022792) investigating a personalized treatment approach to enhance treatment outcomes in CMSP.

**Results:**

Approval was granted by the Ethics Committee II of the University of Heidelberg on August 4, 2020. Recruitment for the microrandomized trial began in May 2021 and is ongoing at the time of submission. By October 10, 2022, a total of 24 participants had been enrolled in the microrandomized trial.

**Conclusions:**

This trial will provide evidence on the feasibility of the PerPAIN app and the initial signals of efficacy of the different intervention components. In the next step, the intervention would need to be further refined and investigated in a definitive trial. This ecological momentary intervention presents a potential method for offering low-level accessible treatment to a wide range of people, which could have substantial implications for public health by reducing disease burden of chronic pain in the population.

**International Registered Report Identifier (IRRID):**

DERR1-10.2196/43376

## Introduction

### Background

Musculoskeletal pain is defined as chronic if it exceeds a time frame of 3 months or recurs for >3 months [1]. Chronic musculoskeletal pain (CMSP) is estimated to affect between 13% and 47% of the population worldwide (eg, in the European Union [2], the United States [3], Canada [4], Australia [5], and low- to middle-income countries [6,7]). The global disease burden caused by CMSP has been increasing by 20.3% in the last 15 years [8]. Therefore, from a public health perspective, there is an urgent need for accessible and effective treatments. To determine the etiology and treatment of chronic pain, it is now widely suggested that a biopsychosocial model be followed [1,9,10]. CMSP is associated with a range of symptoms and disorders, mainly comorbid depression and anxiety [11-17], as well as low self-esteem [18-20], attention to and fear of pain [21,22], and avoidance behaviors [23-25]. Therefore, targeting psychological and behavioral aspects can be effective in treating chronic pain [26,27].

Diary methods including the experience sampling method (ESM) [28,29], where participants are prompted to answer several questionnaires per day during their daily life, have become common for monitoring psychological and somatic symptoms, yielding sufficient response compliance across health conditions and age groups [30]. Pain diaries, specifically paper or electronic versions, have proven high satisfaction, feasibility, and compliance for patients with CMSP, providing good psychometric properties [31-34]. However, using ESM pain diaries that remind participants to rate their level of pain once or several times per day [35] or provide personalized feedback [36] may hold restrictions, as this may promote attention to pain, that is, hypervigilance [37] or attentional bias [38-40], which in turn may even increase pain intensity [41]. This is why it has been suggested to focus on positive psychological processes [42] that may broaden attentional focus to positive aspects in life [43,44] and away from pain.

Similarly, positive interventions may be a strategy to promote a focus on resources and resilience [42,45,46]. Previous studies have shown that positive interventions that aim to increase awareness of positive aspects of life can improve pain intensity and catastrophizing, happiness, positive affect, depression, and anxiety [47-49]. In addition, targeting psychological aspects and comorbidities of chronic pain, cognitive behavioral therapy for low self-esteem has been shown to increase self-esteem, psychological functioning, and depression in populations with transdiagnostic psychiatric disorders [50,51]. Behavioral activation or activity scheduling aimed at increasing positive activities and experiences has shown a large meta-analytic effect in the treatment of depression [52] and first positive evidence in the treatment of chronic pain [53,54].

Following recent advances in digital technology, mobile health (mHealth) approaches such as ecological momentary interventions (EMIs) that deliver psychological treatment components in daily life [55,56] can be a promising way to provide early, low-level, accessible, and scalable treatment [57,58]. As EMIs are provided via mobile phone apps, they may help overcome barriers that can arise for interventions delivered in the clinical setting [28,55,59]. Several studies have shown that app-based approaches are feasible and can be effective in the treatment of chronic pain [60-62]. Furthermore, when patients with chronic pain are offered digitalized monitoring and feedback, they are willing to accept less frequent physician consultations [63].

Within the PerPAIN consortium investigating whether personalized allocation to psychological treatment approaches can enhance treatment effects in CMSP [64], we developed the PerPAIN app. The PerPAIN app targets positive refocusing of attention to positive events using digitalized monitoring, feedback, and microinterventions in daily life. Thus, the PerPAIN app is a complex intervention comprising several intervention components delivered at varying time points.

To evaluate behavioral interventions that comprise multiple intervention components, Collins et al [65] introduced a new approach, that is, the multiphase optimization strategy (MOST) [65,66]. In addition to the preparation and evaluation phases of an intervention, MOST introduces an optimization phase in which individual intervention components can be evaluated separately according to predefined optimization criteria [65,66]. This information can then be used to optimize the intervention as a whole to proceed to the evaluation phase [65,66].

### Objective

In this paper, we describe an optimization trial to evaluate the intervention components of the PerPAIN app in line with MOST [65,66]. Specifically, we designed a microrandomized trial, an experimental research design developed to evaluate and optimize mHealth interventions (for a comprehensive introduction, see Qian et al [67]). By repeatedly randomizing individuals to different forms of an intervention component (ie, within-person randomization), this research design allows the investigation of the immediate effects of intervention components on repeatedly measured proximal outcomes [68], as opposed to distal outcomes measured once at the end of treatment or follow-up. In this microrandomized trial, we aim to (1) establish the feasibility for conducting a microrandomized trial in patients with CMSP (based on acceptability, satisfaction, compliance, adherence, and fidelity of delivering the intervention) and (2) investigate initial signals of efficacy of the feedback component (ie, daily and weekly feedback presented as texts or graphs) and the exercises delivered in microinterventions (namely, the journal of joyful moments, the positive data log, and the activity planner) on the proximal outcomes.

To this end, we hypothesize that feasibility criteria for conducting a microrandomized trial evaluating the PerPAIN app in patients with CMSP will be met (hypothesis 1).

Second, we hypothesize that within participants, momentary absence of pain (candidate primary proximal outcome) after providing feedback (as text or graph) at the end of a training day on which participants reported at least medium (ie, ≥4) absence of pain, positive mood, or subjective activity will, on average across time, be higher compared with time points when no feedback was provided on such days (within-person control condition; hypothesis 2).

Third, we hypothesize that within participants, momentary (a) positive mood and (b) subjective activity (candidate secondary proximal outcomes) after providing feedback (as text or graph) at the end of a training day on which participants reported at least medium (ie, ≥4) absence of pain, positive mood, or subjective activity will, on average across time, be higher compared with time points when no feedback was provided on such days (within-person control condition; hypothesis 3a and 3b).

Finally, we hypothesize that within participants, momentary (a) absence of pain, (b) positive mood, and (c) subjective activity after delivering a microintervention (ie, journal of joyful moments, positive data log, or daily activity planner) at an individually predefined time point will, on average across time, be higher compared with comparable time points on days on which no microintervention was delivered (within-person control condition; hypothesis 4a, 4b, and 4c).

We will also explore differences between the 2 types of feedback presentation and 3 types of microinterventions in terms of efficacy and time trends across the 12-week intervention period. In this paper, we describe the PerPAIN app and its intervention components in detail as well as the microrandomized trial implemented to investigate the feasibility and initial signals of efficacy. For a description of the clinical trial that this microrandomized trial is embedded in, we refer to the protocol by Beiner et al [64].

## Methods

### Procedure, Study Design, and Randomization

A total of 35 patients with CMSP will be recruited to undergo the PerPAIN mobile training, an mHealth app targeted at refocusing of attention on positive events, comprising digitalized monitoring using the ESM, personalized feedback, and EMIs. During a one-on-one structured briefing session, the participants will receive the study app preinstalled on a study smartphone. A trained member of the study team will explain the basic functionalities of the device and app to the participant and answer any potential questions before the participant takes the device into their daily life. The app sends different types of prompts each day for 12 weeks, including monitoring questionnaires, feedback presentations, and microinterventions. Answering all prompts requires approximately 15 minutes per day. A trained member of the study team checks in with the participants up to 6 times to answer any potential questions. The app follows a microrandomized design to evaluate the intervention components (see Multimedia Appendix 1 for a visualization). The training follows a fixed schedule across 12 weeks and entails 4 types of randomization procedures, resulting in a total of *t*=143 decision points, that is, time points when automated randomization of an intervention component takes place in the backend of the app. First, every evening during the 12 weeks (84 decision points), participants will receive microrandomized daily feedback (text vs graph vs none). Second, at the end of each training week (12 decision points), the participants receive microrandomized weekly feedback (text vs graph vs none). Third, in the second half of the training, the exercises participants are prompted to do as microinterventions are randomized (journal of joyful moments vs positive data log vs daily activity planner vs none) every day for 6 weeks (42 decision points). Fourth, starting from week 8, the weekly activity planner is randomized (on vs off) each week for the remaining 5 weeks (5 decision points). The ESM monitoring questionnaires prompted 5 times per day to serve as proximal outcome assessments. This microrandomized trial is nested in and carried out as part of the PerPAIN trial (see the protocol by Beiner et al [64] for a detailed description of this study, adhering to the Standard Protocol Items: Recommendations for Interventional Trials 2013 checklist [69]).

### Participants

For this microrandomized trial, we aim to recruit adult patients (aged ≥18 years) experiencing CMSP for >3 months as a symptom of nonspecific chronic back pain, osteoarthritis, fibromyalgia syndrome, or rheumatoid arthritis. Participants must be able to see and use a mobile phone (including visual aids). Exclusion criteria include insufficient or unclear treatment of the underlying disease; severe visual impairment or inability to use a mobile phone; application for retirement or pension pending; ongoing psychotherapy; and severe physical, psychiatric, or neurological comorbidity (see the protocol by Beiner et al [64] for more details). Participants will be recruited primarily via an outpatient pain clinic.

### PerPAIN App

#### Overview

The PerPAIN app is a stand-alone, app-based, self-help training with a total duration of 12 weeks, entailing 3 core components. First, it uses the ESM to monitor participants’ momentary absence of pain, positive mood, subjective activity, and context several times a day in a positive pain diary (ie, monitoring). Second, the app provides personalized feedback to the participants on the basis of the monitoring data. Third, short exercises based on the principles of cognitive behavioral psychotherapy and positive psychology are provided, which are both taught and implemented within the daily life context (ie, microinterventions). Among these components, gamification elements are used to potentially increase compliance and user engagement.

#### Monitoring and Feedback

The ESM will be used to collect entries for a positive pain diary. The app will prompt participants to answer 4 monitoring questionnaires per day scheduled at semirandom time points within set blocks of time during their waking hours to record their momentary experiences and context. The ESM variables, that is, sleep quality, trust in the intervention, absence of pain, positive mood, and subjective activity, as well as appraisals of momentary and daily context, are rated on a 7-point scale. The monitoring questionnaire consists of 16 to 21 items (depending on the type of prompt and branching rules; see Multimedia Appendix 2 for a full list of items).

The PerPAIN app uses the monitoring data to provide feedback on the three main ESM monitoring and outcome variables: (1) absence of pain, (2) positive mood, and (3) subjective activity. We implemented three types of feedback: (1) immediate feedback presented directly after a monitoring questionnaire, (2) daily feedback presented after the last monitoring questionnaire of the day, and (3) weekly feedback presented after each week of monitoring. In accordance with our aim of refocusing of attention on positive events, immediate and daily feedback is only provided if it qualifies as *positive*. Therefore, immediate and daily feedback on each variable is only displayed if the (mean) score exceeds a predefined threshold of 4, that is, the center of the 7-point scale.

Immediate feedback is designed as a type of gamification. After the participant completes filling out the monitoring questionnaire, the conditions for providing positive feedback are checked. Whenever a variable is ≥4, meaning the participant is considered to be pain-free, in a good mood, or active, feedback is displayed at the end of the questionnaire, that is, the participant collects a pain-free, feel-good, or activity point, respectively (Multimedia Appendix 3).

Daily feedback is made available to the participant in a separate prompt after the last random monitoring questionnaire of the day. Before triggering a prompt for daily feedback, a server checks the conditions for providing positive feedback. Whenever the daily mean score of at least 1 of the 3 main ESM variables is ≥4, daily feedback is generated. If, at the same time, one of the other mean scores is <4, feedback for this variable is masked out. For example, if the mean scores of positive mood and subjective activity are both 5 and the mean score of absence of pain is 2, daily feedback is generated, but no value is presented for the absence of pain. Whenever daily feedback is available, a stratified randomization procedure is applied to determine the type of feedback presentation (1:1:1). Feedback is either presented as a bar chart or as a short text, or, in a control condition, no daily feedback is provided. Immediately after the feedback prompt, a monitoring questionnaire containing only the questions regarding the main ESM outcome variables is presented to assess proximal, that is, immediate effects of the daily feedback component.

Weekly feedback is made available to the participant in a separate notification after each week of monitoring, that is, in the morning of every 7th day at the earliest possible time for the app to interact with the participant. Unlike daily feedback, weekly feedback is always provided without any restrictions concerning the mean scores of the variables, that is, values <4 are also displayed, as this matches most accurately a paper-pencil positive pain diary that was piloted before this study. Again, a stratified randomization procedure is applied to set the type of feedback presentation (1:1:1). Weekly feedback is either presented as a bar chart or a short text (Multimedia Appendix 3), or, in a control condition, no weekly feedback is provided. Immediately after the feedback prompt, a monitoring questionnaire containing only the questions regarding the main ESM outcome variables is presented to assess the proximal effects of the weekly feedback component.

#### Microinterventions

**Figure 1:**
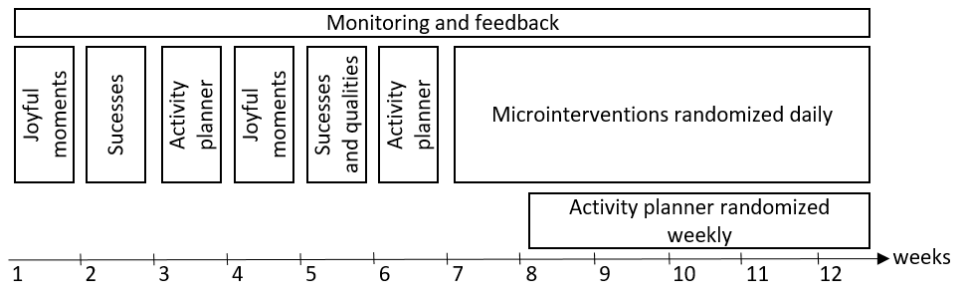
Timeline of the PerPAIN training intervention components.

In addition to the monitoring and feedback components, the PerPAIN app incorporates the principles of EMI. Specifically, it entails 3 exercises implemented as microinterventions alternating across the 12-week training period (Figure 1). In the first half of the training, participants are prompted to complete 1 exercise presented as a microintervention per week. As previously used in an EMI targeted at ecological translation of principles of compassion-focused therapy to daily life [70], the app will offer enhancing, consolidating, and interactive microinterventions. Initially, each exercise is introduced to the participant in an introduction block at the beginning of a new week (ie, enhancing). Once familiar with the microintervention, the participants set the time they wish to be prompted to complete the exercise once a day in a separate notification (ie, consolidation). In addition, if indicated by the participants’ answers on a monitoring questionnaire, an exercise is prompted interactively. In the second half of the training, starting from week 7, consolidating and interactive microinterventions alternate each day according to the randomization procedure described in the *Procedure, Study Design, and Randomization* section.

The journal of joyful moments is based on a positive intervention called the “3 good things” intervention [71]. In a continuously growing list structured like a diary, participants collect moments that were happy or joyful for them; things that made them smile; or, in general, made them feel good. The aim of this technique is to draw attention to positive things that happen in daily life, increase well-being, and decrease feelings of depression [71]. Participants can make a new entry (“Please note your joyful moment”) once per day at the consolidation prompt, manually whenever they wish to make an entry, or during a monitoring prompt whenever they are likely to have experienced a joyful moment, according to their answers in a monitoring questionnaire. For this purpose, the following conditions were defined: (1) the participant indicates that they are in a good mood, that is, ≥4; (2) the participant indicates that their absence of pain is high, that is, ≥4; and (3) the participant indicates a high level of activity, that is, ≥4 (Multimedia Appendix 4). Once the enhancing exercise is completed, the participant can (in all following training weeks) make an entry in the journal and look at the growing list of joyful moments from the home screen of the app (Multimedia Appendix 5).

The positive data log is based on an intervention manual developed to increase self-esteem [72]. The aim of a positive data log is to help correct information-processing errors [73]. The microintervention consists of two parts: (1) collecting small successes during daily life and (2) inferring positive qualities of oneself from the list of successes. When this microintervention is first introduced in week 2, participants collect small or large successes they have experienced in their daily life in a diary. A success can be anything positive or successful the participant does, everything they do well, and anything that they made progress with or put effort into. The aim of this technique is to draw attention to one’s own small or big successes in daily life to build self-efficacy and confidence instead of focusing on things that go wrong [72]. Participants can make a new entry (“Please note your success”) (1) once per day at the consolidation prompt, (2) manually whenever they wish to make an entry, or (3) during a monitoring prompt whenever they are likely to have had a success according to their answers in a monitoring questionnaire or whenever they do not feel well and will likely benefit from redirecting their attention to something positive. For this purpose, the following conditions were defined: (1) the participant indicates that they have experienced a positive event, (2) the participant indicates that they are at work, school, or university to uncover work-, school-, or university-related successes, and (3) the participant indicates that they are in a bad mood or in pain (Multimedia Appendix 4).

In week 5 of the training, when this microintervention is repeated (Figure 1), the section on positive qualities is added to the positive data log. After collecting several successes in their daily life, that is, observable behavior, participants are guided to infer positive qualities that underlie those successful behaviors by answering the question “What qualities does a person have that shows these behaviors?” Thus, participants start a second list with their own positive qualities. Each time the participant makes an entry into their positive data log, they are invited to add a positive quality. Once the enhancing of each part of the exercise is completed, the participant can (in all following training weeks) make an entry to the positive data log and look at the growing list of successes and positive qualities from the home screen of the app (Multimedia Appendix 5).

The activity planner is based on a manual for behavioral activation [74], a behavioral therapy technique used in the treatment of depression [75] and chronic pain [76]. In behavioral activation, pleasant activities are systematically increased to change corresponding cognitions. In an introductory block, the participant is educated about the interdependence between pleasant activities and positive thoughts and feelings (Multimedia Appendix 5). The participant then creates their own activity plan for the week in a guided process. From a list of activities from different life domains, that is, sociability, hobbies and free time activities, sports and physical activities, culture and education, nature and gardening, and self-care and delight, the participant is invited to choose 3 activities or insert activities they would like to do in the following week. After choosing each activity, the participant is instructed to define the number of times they would like to do the activity. This step is based on the idea that progress toward specific goals can be monitored easily and can therefore be more motivating [74]. During the activity week, the participant can monitor their progress with their planned activities. Each morning, the participant receives a notification reminding them of their planned activities. Optionally, they can plan an additional activity for the upcoming day only (ie, daily activity planner). Participants can make a new entry (“Please tick off all activities you have done today”) once per day at the consolidation prompt or manually by entering the activity planner from the home screen of the app. On the last consolidation prompt of the activity week, the planned and actual frequencies of each activity are presented to the participant, and they can indicate whether they have achieved, exceeded, or not accomplished their goal. The achievement or exceedance of goals is rewarded using the gamification elements described in the *Gamification* section.

#### Gamification

Gamification is a concept that integrates game elements into nongame contexts. It builds on the engaging and motivating potential of games [77]. In mHealth, gamification elements can be used to increase compliance and shape and reward desired behaviors [78]. After downloading an mHealth app, user engagement can vary greatly (eg, see the study by Rahman et al [79]), which is why it is a common objective of researchers to design apps to optimally support engagement [80]. In the PerPAIN app, we implemented three gamification elements aimed at increasing compliance and providing rewards: (1) an overall training score, (2) immediate feedback designed as points, and (3) a progress bar. The overall training score increases whenever the participant answers a prompt or makes an entry (Multimedia Appendix 3). Hence, this gamification element is aimed at increasing users’ overall engagement with the app. In the overall introduction to the app as well as the enhancing blocks introducing the microinterventions, the participant is informed which actions lead to an increase in the training score (eg, answering a monitoring prompt, making an entry in the journal of joyful moments, or achieving an activity goal). After a monitoring prompt, the participant can receive 3 types of points (ie, pain-free, feel-good, and activity points), whenever the values of the 3 scales were ≥4. These points are collected throughout the training and mainly serve as positive feedback but may also reinforce behaviors that lead to reporting higher values of absence of pain, positive mood, or subjective activity in the monitoring questionnaire. The overall user advancement is presented to the participant on a summary page that includes a progress bar displaying progression throughout the training using an icon of a person walking on a path of 12 tiles representing the 12 training weeks. This gamification element aims to increase the transparency and completion rates of the training.

### Measures

We will assess the feasibility of delivering the smartphone training and the effects of intervention components.

#### Feasibility Measures

In line with previous research on the feasibility of delivering EMIs [70,81,82], feasibility measures will include acceptability, intervention adherence, and fidelity. Acceptability will be assessed in a debriefing questionnaire rating satisfaction with the intervention, ease of use, accessibility, and comprehensiveness [70,81,82]. In addition, we will use the user version of the mobile app rating scale [83] to assess the subjective quality of the app. Adherence to the mobile training will be operationalized by the completion rates of the intervention components (ie, mean number of monitoring questionnaires, consolidating or interactive tasks completed/wk, and [mean] number of feedback prompts answered). As a core feature of the PerPAIN training is the delivery of intervention components in daily life, that is, in varying contexts, aiming to support a positive attention focus while reducing transfer effects, we will measure fidelity by the variety of contexts in which participants engage with the app and the proportion of participants who still use the app at the end of the 12-week training period. To establish levels of feasibility, we will classify our feasibility outcomes using a traffic light system into 3 categories, that is, green=feasibility fully established, yellow=feasibility established with a need for modification of the study procedures, and red=feasibility not established [70] (Table 1).

**Table 1:** Feasibility criteria of delivering the PerPAIN training.

Category	Acceptability	Adherence	Fidelity
Red (feasibility not established)	Low satisfaction in the debriefing questionnaire (ie, mean satisfaction rating of ≤3 on a 7-point scale) and the uMARS^a^ (ie, mean subjective quality rating of ≤2 on a 5-point scale)	Poor compliance to the monitoring and feedback component (≥60% of the participants answering on average <50% of the questionnaires/d and ≥60% of the participants viewing their provided feedback on average less than once/wk) and EMI^b^ tasks (ie, mean number of consolidating or interactive EMI tasks completed/wk is <0.8)	Poor use of the app overall (≥60% of the participants using the app for <3 wk) and poor variability of intervention contexts (≥60% of the participants using the app in 1 context exclusively, as measured by the “current activity” item)
Yellow (feasibility established but study procedures need modifying)	Fair satisfaction in the debriefing questionnaire (ie, mean satisfaction rating of >3 on a 7-point scale) and the uMARS (ie, mean subjective quality rating of >2 on a 5-point scale)	Fair compliance to the monitoring and feedback component (≥60% of the participants answering on average at least 50% of the questionnaires and ≥60% of the participants viewing their provided feedback on average at least once/wk) and EMI tasks (ie, mean number of consolidating or interactive EMI tasks completed/wk is ≥0.8)	Fair use of the app overall (≥60% of the participants using the app for ≥3 wk) and fair variability of intervention contexts (≥60% of the participants using the app in at least 2 contexts, as measured by the “current activity” item)
Green (feasibility fully established)	Moderate to high satisfaction in the debriefing questionnaire (ie, mean satisfaction rating of >4 on a 7-point scale) and the uMARS (ie, mean subjective quality rating of >3 on a 5-point scale)	Moderate to strong compliance to the monitoring and feedback component (≥80% of the participants answering at least 50% of the questionnaires and ≥80% of the participants viewing their provided feedback on average at least once/wk) and EMI tasks (ie, mean number of consolidating or interactive EMI tasks completed/wk ≥1)	Moderate to high use of the app overall (≥80% of the participants using the app for ≥ 6 wk) and moderate to high variability of intervention contexts (≥80% of the participants using the app in at least 2 contexts, as measured by the “current activity” item)

^a^uMARS: user version of the mobile application rating scale.

^b^EMI: ecological momentary intervention.

#### Candidate Primary Outcome

The candidate primary outcome of the microrandomized trial is the proximal measure of the absence of pain assessed in the monitoring questionnaires. Specifically, we will investigate the immediate effect of the daily feedback component on the absence of pain rating measured on a 7-point scale in the monitoring questionnaire presented either directly after daily feedback was displayed or in a separate prompt without feedback being displayed (control condition), depending on the microrandomization (Multimedia Appendix 1).

#### Candidate Secondary Outcomes

The 2 candidate secondary outcomes of the microrandomized trial are the proximal measures of positive mood and subjective activity assessed on a 7-point scale in the monitoring questionnaires. As for the candidate primary outcome, we will use the ratings given in the monitoring questionnaire presented either directly after daily feedback was displayed or in a separate prompt without feedback being displayed (control condition), depending on the microrandomization.

#### Time-Fixed Covariates

In line with the PerPAIN study [64], we will use the treatment condition (personalized vs nonpersonalized treatment allocation), gender, and baseline pain severity (as measured by the German version of the Multidimensional Pain Inventory [84]) as covariates in all models.

### Sample Size

This microrandomized trial forms part of the PerPAIN randomized controlled trial [64]. Given that we expect to include 35 participants in this microrandomized trial, we performed power analysis for microrandomized trials [85] to determine the effect sizes we will be able to detect in the given study setup with sufficient power of at least 80% testing at α=.05 (for a description of the tool, see Seewald et al [86]).

Regarding the daily feedback component with 1 decision time point for a total of 84 days, we will be able to detect an average proximal treatment effect of 0.16 with a power of 80.4% and a sample size of 35, assuming that at least 1 ESM variable is >4 (therefore, available for feedback) on every second day of the study (ie, availability parameter of 0.5). Regarding the microinterventions with 1 decision time point for a total of 42 days, we will be able to detect an average proximal treatment effect of 0.18 with a power of 80.8%, a sample size of 35, and an availability parameter of 1, as microinterventions will be prompted every day without any restrictions. Intervention components randomized weekly (weekly feedback and weekly activity planner) do not produce sufficient power to be tested for proximal treatment effects in this study.

### Assessment of Safety in the Microrandomized Trial

Throughout participation in this microrandomized trial, we will monitor any serious adverse events, such as serious incidents resulting in death or hospitalization, and adverse events (see the protocol by Beiner et al [64] for more details). Furthermore, we will record any potential adverse device effects and technical problems reported by participants, such as crashing of the app or bugs. Whenever possible, we will fix technical problems immediately and introduce a new version of the app for future participants using the app.

### Statistical Analysis

To address hypothesis 1, we will use descriptive statistics and 95% CIs as appropriate to evaluate the feasibility of delivering the PerPAIN training in line with the predefined feasibility criteria described in Table 1.

To test initial signals of efficacy on candidate primary and secondary proximal outcomes, we will use the weighted and centered least squares estimator to estimate the causal excursion effect of the daily feedback component [87,88]. To investigate whether there is an overall effect (ie, on average across time) of daily feedback on our candidate primary proximal outcome momentary absence of pain (hypothesis 2), we will calculate the weighted and centered least squares estimator by specifying a generalized estimation equation with absence of pain in the subsequent monitoring questionnaire as the proximal outcome, with a treatment indicator specifying whether daily feedback was delivered (=1) or not (=0), including 84 decision points, that is, once every day for 12 weeks. As feedback is only delivered whenever the daily average of one of the main outcome variables was >4, we will create a new variable *good_day* (1=at least 1 outcome is >4 and 0=no outcome is >4) serving as an availability indicator in the equation. The analysis will be controlled for time-fixed covariates. To address hypotheses 3a and 3b, we will repeat this procedure using our candidate secondary proximal outcomes: (a) momentary positive mood and (b) momentary subjective activity at the subsequent monitoring questionnaire as the proximal outcome. In the next step, to explore the effect of different feedback types individually (ie, as text or graph), we will include 1 treatment indicator for each type of feedback. Furthermore, we will explore whether there is a time trend in the effects of daily feedback by including a variable *training_day* counting the days in the study (with the value 0 on the first day).

To investigate the immediate effects of the microinterventions, we will use momentary absence of pain, positive mood, and subjective activity rated on a 7-point scale in the next monitoring questionnaire prompted after the predefined consolidation time, unless the consolidation prompt was the last prompt of the day, to avoid overnight effects. To determine whether there is an overall effect, that is, on average across time, of the microinterventions (hypotheses 4a, 4b, and 4c), we will specify 3 generalized estimation equations with a treatment indicator specifying whether a microintervention was delivered at the individually predefined consolidation time on that day (=1) or not (=0), that is, control condition, using each outcome: (a) momentary absence of pain, (b) momentary positive mood, and (c) momentary subjective activity at the subsequent prompt as a proximal outcome, including 42 treatment occasions, that is, once every day between week 7 and 12. There will be no availability indicator, and time-fixed covariates will be included. We will then include 1 treatment indicator for each type of intervention to investigate the effects of the 3 microinterventions individually (ie, journal of joyful moments, positive data log, and daily activity planner) and explore the time trend in the effects of the microinterventions by including the *training_day* indicator. Data analysts will be blinded to the treatment indicators.

## Results

The Ethics Committee II of the University of Heidelberg approved this study (2020-579N) on August 4, 2020. At the time of submission of this protocol (October 10, 2022), we are working with trial protocol version 6 (October 6, 2021). The outcome assessments are ongoing. The first enrollment in this microrandomized trial was conducted in November 2021. As of October 10, 2022 (the date of submission), 24 participants were enrolled in the microrandomized trial, and 13 participants completed the training. Data will not be accessed for interim analysis before the completion of all outcome assessments. The last assessment for the last participant is scheduled for July 2023, and we expect the results to be published in early 2024.

## Discussion

Extending the widely used method of symptom monitoring using pain diaries, we will investigate the feasibility and initial signals of efficacy of a newly developed self-help app targeted at refocusing of attention to positive events with different intervention components based on principles of EMI. EMIs offer the great advantage of delivering intervention components in various contexts in daily life [28,55-59]. EMIs are frequently implemented as hybrid interventions [70,81,82] or stand-alone interventions, such as the PerPAIN app. Owing to the microrandomized design, we will be able to evaluate individual intervention components separately to optimize the intervention as a whole and proceed to the evaluation phase in line with MOST [65,66]. The results of this optimization trial will show whether delivering an app-based self-help training with different intervention components is feasible. Furthermore, we will investigate its acceptability, satisfaction, compliance, adherence, and fidelity in a population of patients with CMSP. The results of this trial promise first insights into the potential proximal effects of delivering daily feedback and prompting microinterventions to improve the absence of pain and increase positive mood and subjective activity. The results of this trial may suggest ways to optimize these intervention components. For instance, the results may point toward 1 type of feedback presentation (eg, as a graph) or microintervention (eg, activity planner) having greater initial effects than another. Alternatively, there may be initial evidence of a time trend across the 12 weeks of the training, which could inform the setup of future studies.

If this trial is successful, the next step will be to proceed to a confirmatory trial with a larger sample powered to detect the expected effect sizes established in this trial.

However, for the interpretation of the results of this study, it should be considered that the effect sizes that will be established may be diminished because of the design of the PerPAIN study this microrandomized trial is nested in. As a result of the randomization procedure to investigate a personalized treatment approach (see the protocol by Beiner et al [64]), 50% (17/35) of the participants in this microrandomized trial will be allocated to participate in the PerPAIN training according to a personalized treatment allocation procedure, whereas the other half will be allocated to the PerPAIN training against the personalization approach.

Positive psychological approaches seem to be promising for the treatment of CMSP [42,45-49]. Therefore, investigating how these approaches can be implemented using mHealth technologies is an important step in tackling the global disease burden caused by CMSP. Overall, EMIs using microinterventions offer a great opportunity to provide low-level treatment in the context of daily life, thereby overcoming barriers to interventions delivered in the clinical context [55-57,59]. After positive scientific evaluation, this type of treatment promises significant public health impact as it is scalable to a large population of patients with CMSP, which may ensure continuity of care and reduce on-site consultations [63].

## Appendix

Multimedia Appendix 1 Visualization of the microrandomized design.

Multimedia Appendix 2 Full list of the monitoring items.

Multimedia Appendix 3 Examples of immediate (left) and daily (middle) feedback and gamification (right) in the PerPAIN app.

Multimedia Appendix 4 Conditions for interactive microinterventions prompted within a monitoring questionnaire.

Multimedia Appendix 5 Examples of microinterventions in the PerPAIN app (from left to right: journal of joyful moments, positive data log, and activity planner).

## Abbreviations

CMSP: chronic musculoskeletal pain
EMI: ecological momentary intervention
ESM: experience sampling method
mHealth: mobile health
MOST: multiphase optimization strategy
