# Supplementary material for: Refocusing of Attention on Positive Events Using Monitoring-Based Feedback and Microinterventions for Patients With Chronic Musculoskeletal Pain in the PerPAIN Randomized Controlled Trial: Protocol for a Microrandomized Trial
Source: JMIR Res Protoc. 2023 Sep 20;12:e43376. doi: 10.2196/43376 (PMC10551789; doi:10.2196/43376)
Supplement: Multimedia Appendix 1 [file resprot_v12i1e43376_app1.docx]

Figure S1. Visualization of the micro-randomized design.^a^


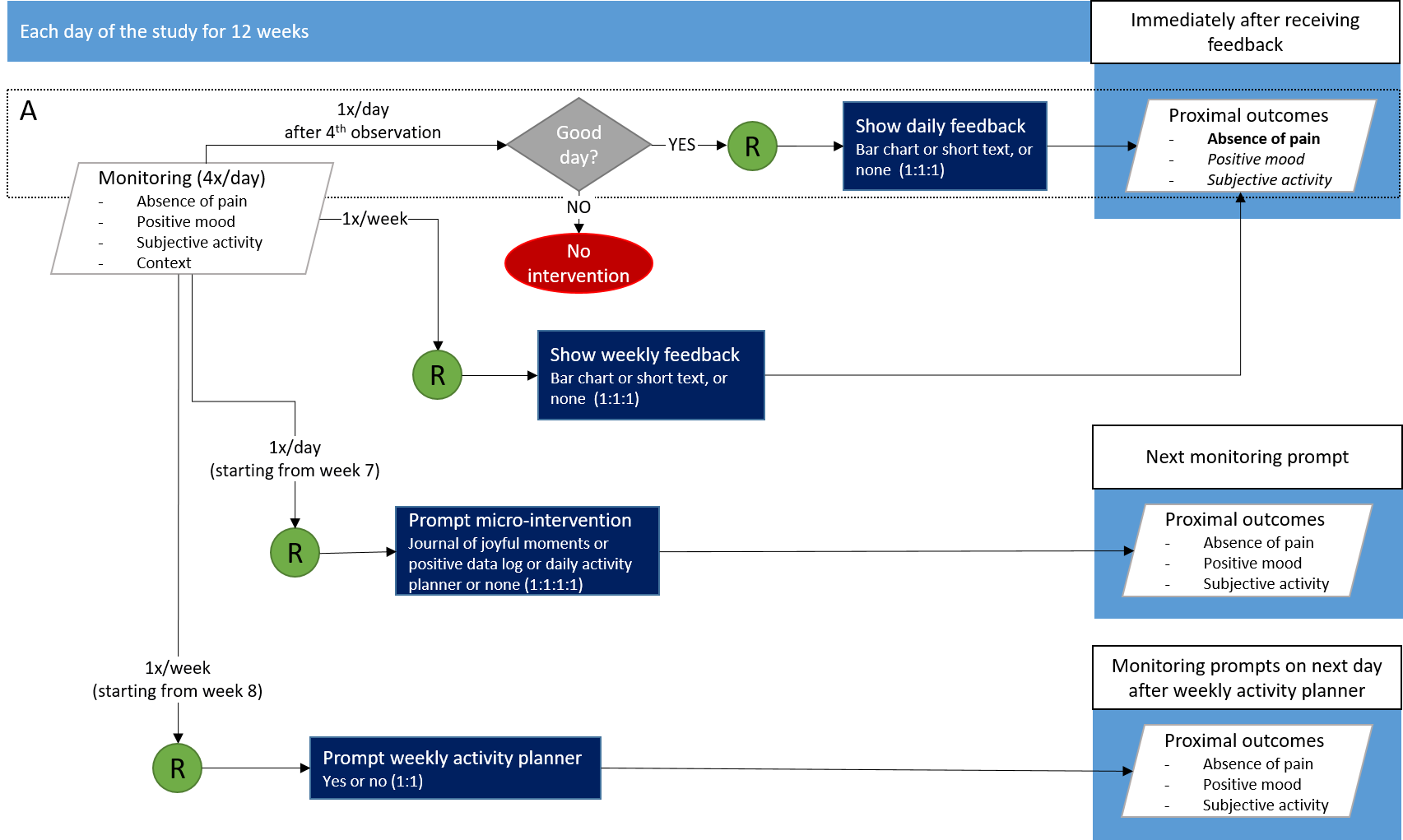


Note. Box A shows candidate primary proximal outcome “Absence of pain” after receiving daily feedback (in bold), and candidate secondary proximal outcomes positive mood and subjective activity after receiving daily feedback (in italics). R = randomization

^a^Illustration style based on <http://people.seas.harvard.edu/~samurphy/JITAI_MRT/mrts4.html> (accessed on October 10, 2022)
