# Supplementary material for: Refocusing of Attention on Positive Events Using Monitoring-Based Feedback and Microinterventions for Patients With Chronic Musculoskeletal Pain in the PerPAIN Randomized Controlled Trial: Protocol for a Microrandomized Trial
Source: JMIR Res Protoc. 2023 Sep 20;12:e43376. doi: 10.2196/43376 (PMC10551789; doi:10.2196/43376)
Supplement: Multimedia Appendix 2 [file resprot_v12i1e43376_app2.docx]

Table S1. Full list of monitoring items.

| Scale | Item | Response options |
| --- | --- | --- |
| Sleep quality | I slept well last night.^a^ | 1 = not at all, 7 = completely |
| Trust in intervention | I have confidence in this treatment.^a^ | 1 = not at all, 7 = completely |
| Absence of pain | I am painfree. | 1 = not at all, 7 = completely |
| Positive mood | I am in a good mood. | 1 = not at all, 7 = completely |
|  | I feel relaxed. | 1 = not at all, 7 = completely |
|  | I feel satisfied. | 1 = not at all, 7 = completely |
|  | I feel cheerful. | 1 = not at all, 7 = completely |
| Subjective activity | I feel fit. | 1 = not at all, 7 = completely |
|  | I feel active. | 1 = not at all, 7 = completely |
|  | I have been physically active since the last prompt. | 1 = not at all, 7 = completely |
| Current activity | What am I doing at the moment? | 1 = work/ university/ school,  2 = resting/ relaxing,  3 = housework/ cooking,  4 = shopping/ errands,  5 = eating/ drinking,  6 = internet/ social media/ phone/ computer,  7 = TV/ streaming,  8 = reading,  9 = having a conversation,  10 = hobby (no physical activity),  11 = physical activity/ exercising,  12 = going out,  13 = doctor’s appointment/ physiotherapy/ medical care,  14 = nothing in particular,  15 = other |
|  | What other activity?^b^ | [Free input] |
|  | This is pleasant. | 1 = not at all, 7 = completely |
|  | This is easy for me. | 1 = not at all, 7 = completely |
|  | I am satisfied with this activity. | 1 = not at all, 7 = completely |
| Current social context | I am alone. | 1 = agree, 2 = disagree |
|  | Who am I with?^c^ | 1 = partner,  2 = family,  3 = friend/s,  4 = colleagues/ classmates,  5 = other acquaintances,  6 = strangers,  7 = pet/s,  8 = someone online/ on the phone |
|  | Who am I with online/ on the phone?^d^ | 1 = partner,  2 = family,  3 = friend/s,  4 = colleagues/ classmates,  5 = other acquaintances,  6 = strangers |
|  | This is pleasant. | 1 = not at all, 7 = completely |
|  | Please think of the most important event since the last prompt. |  |
| Current event | The event was pleasant. | 1 = not at all, 7 = completely |
|  | The event was positive. | 1 = not at all, 7 = completely |
| Everyday life | My everyday life was easy for me today.^e^ | 1 = not at all, 7 = completely |

^a^Item only displayed in morning questionnaire.
^b^Item only displayed if previous question was answered “other”.
^c^Item only displayed if previous question was answered “agree”.
^d^Item only displayed if previous question was answered “someone online/ on the phone”.
^e^Item only displayed in evening questionnaire.
