# Supplementary material for: Refocusing of Attention on Positive Events Using Monitoring-Based Feedback and Microinterventions for Patients With Chronic Musculoskeletal Pain in the PerPAIN Randomized Controlled Trial: Protocol for a Microrandomized Trial
Source: JMIR Res Protoc. 2023 Sep 20;12:e43376. doi: 10.2196/43376 (PMC10551789; doi:10.2196/43376)

Figure S2. Examples of immediate (left) and daily (middle) feedback, and gamification (right) in the PerPain-app.

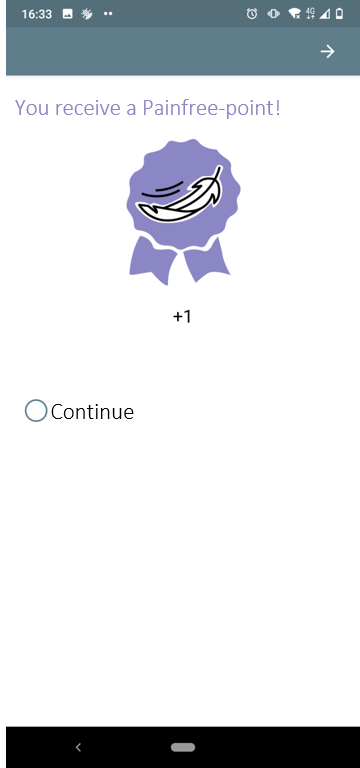

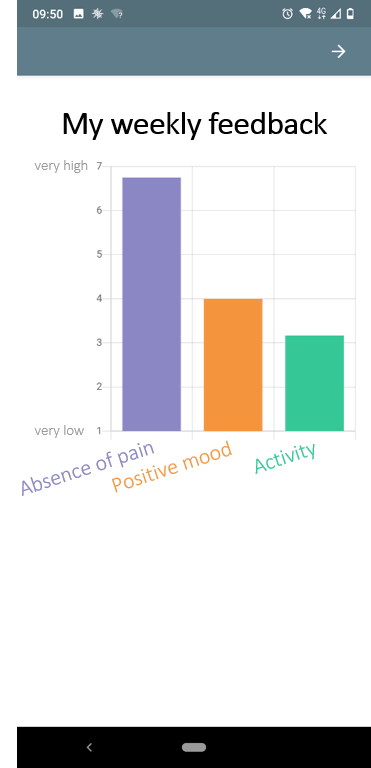

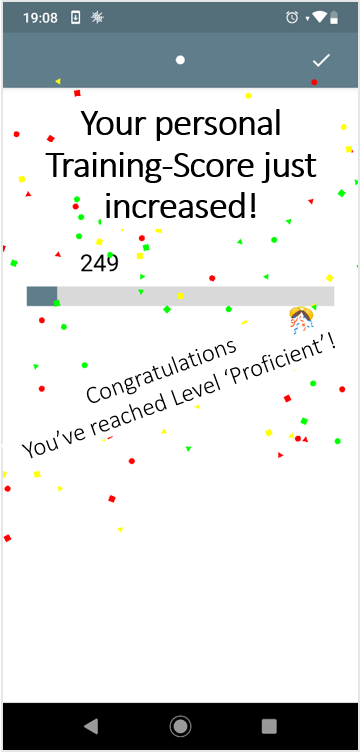

Supplement: Multimedia Appendix 3 [file resprot_v12i1e43376_app3.docx]
