# Supplementary material for: Refocusing of Attention on Positive Events Using Monitoring-Based Feedback and Microinterventions for Patients With Chronic Musculoskeletal Pain in the PerPAIN Randomized Controlled Trial: Protocol for a Microrandomized Trial
Source: JMIR Res Protoc. 2023 Sep 20;12:e43376. doi: 10.2196/43376 (PMC10551789; doi:10.2196/43376)
Supplement: Multimedia Appendix 4 [file resprot_v12i1e43376_app4.docx]

Table S2. Conditions for interactive micro-interventions prompted within a monitoring questionnaire.

| Micro-intervention | Condition^a^ |
| --- | --- |
| Journal of joyful moments | “What am I doing at the moment?” selected as “physical activity/ exercising” AND “I am in a good mood.” > 4 AND (“I feel relaxed.” + “I feel satisfied.” + “I feel cheerful.”) / 3 > 4  “I am painfree” > 4 AND NOT “What am I doing at the moment?” selected as “physical activity/ exercising”  “I am in a good mood.” > 4 AND (“I feel relaxed.” + “I feel satisfied.” + “I feel cheerful.”) / 3 > 4 AND NOT “What am I doing at the moment?” selected as “physical activity/ exercising”  (“I feel fit.” + “I feel active.”) / 2 > 4 OR “I have been physically active since the last prompt.” > 4 AND NOT “What am I doing at the moment?” selected as “physical activity/ exercising” |
| Positive data log | “The event was positive.” > 4  “What am I doing at the moment?” selected as “work/ university/ school”  “I am in a good mood.” < 4 OR “I am painfree.” < 4 |

^a^Bullet points are OR-conditions.
