# Supplementary material for: Refocusing of Attention on Positive Events Using Monitoring-Based Feedback and Microinterventions for Patients With Chronic Musculoskeletal Pain in the PerPAIN Randomized Controlled Trial: Protocol for a Microrandomized Trial
Source: JMIR Res Protoc. 2023 Sep 20;12:e43376. doi: 10.2196/43376 (PMC10551789; doi:10.2196/43376)

Figure S3. Examples of micro-interventions in the PerPain-app (from left to right: journal of joyful moments, positive data log, activity planner).

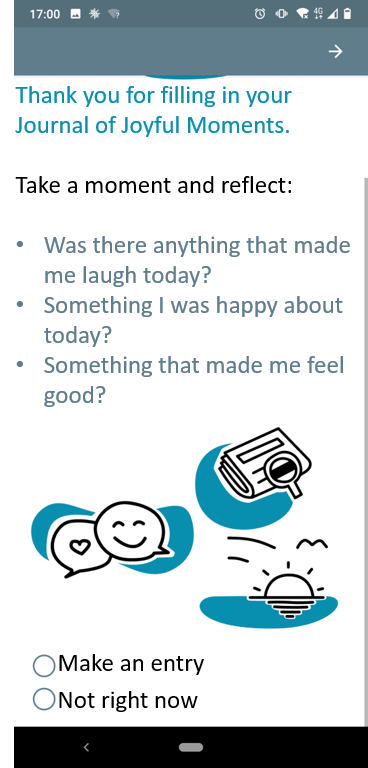

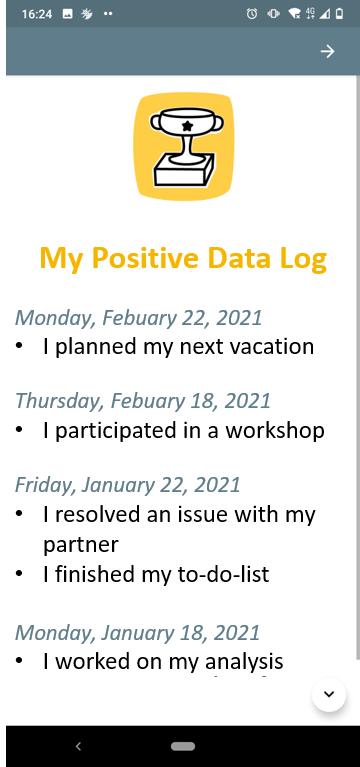

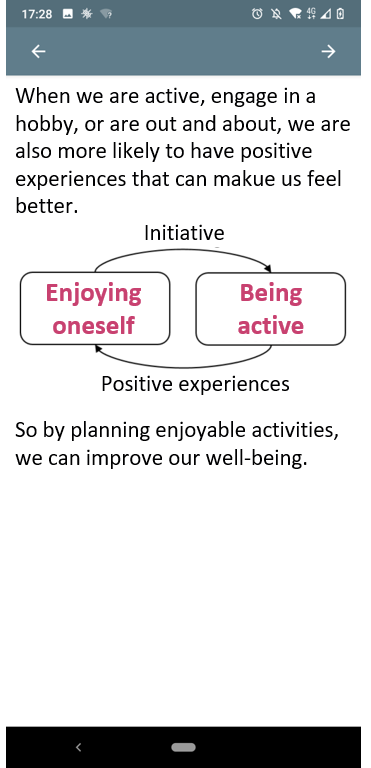

Supplement: Multimedia Appendix 5 [file resprot_v12i1e43376_app5.docx]
